# Assessing Genetic Variability and Population Structure of *Alnus glutinosa* (Black Alder) in Kazakhstan Using SSR Markers

**DOI:** 10.3390/plants13213032

**Published:** 2024-10-30

**Authors:** Aidana Nurtaza, Damira Dyussembekova, Alexandr Shevtsov, Symbat Islamova, Indira Samatova, Saule Koblanova, Olga Borodulina, Almagul Kakimzhanova

**Affiliations:** 1 National Center for Biotechnology, 13/5, Korgalzhyn Road, Astana 010000, Kazakhstan; nurtazaaidana6@gmail.com (A.N.); dussembekova.damira@gmail.com (D.D.); ncbshevtsov@gmail.com (A.S.); islamovasimbat@gmail.com (S.I.); samatova112@gmail.com (I.S.); saule200707@inbox.ru (S.K.); 2 Department of Natural Sciences, Kostanay Regional University, 47 Baytursynov Str., Kostanay 110000, Kazakhstan; jury63@mail.ru

**Keywords:** *Alnus glutinosa*, SSR, genetic diversity, population structure

## Abstract

*Alnus glutinosa* plays a crucial role in flood control, riverbank stabilization, and water purification. Recognized for its ecological significance, it is listed in the Red Book of Kazakhstan. This study investigated the genetic variability of *A. glutinosa* populations in Kazakhstan, analyzing 78 trees from seven populations in the Bayanaul mountain forest massif and the northern Turgay regions using 12 SSR markers. The study identified an average of 6.3 alleles and 2.783 effective alleles, as well as observed and expected heterozygosities of 0.570 and 0.562, respectively, reflecting genetic diversity. Among the populations, KS1 (northern Turgay) and PVL3 (Bayanaul) displayed the highest diversity, while PVL5 (Bayanaul) showed slightly lower diversity. The analysis of molecular variance results indicated that 86% of the genetic diversity occurred within populations, with 14% attributed to differences between populations. A UPGMA tree based on Nei’s genetic distance revealed three distinct clusters, suggesting geographically structured genetic variability in *A. glutinosa* populations.

## 1. Introduction

The genus *Alnus* P. Miller (alder) comprises deciduous trees that can reach up to 30 m in height, as well as smaller shrubs (1–1.5 m), all belonging to the Betulaceae family. The species are distributed widely across the temperate forests of the Northern Hemisphere, including Eurasia and North America [1,2].

Currently, over 30 species of the subgenus *Alnus* are recognized, with *Alnus glutinosa* (L.) Gaertn. (2*n* = 28), commonly known as black or sticky alder, being the most well known and widespread. This actinorhizal species thrives particularly well in wet environments [3,4]. *A. glutinosa* is of particular interest as a pioneer species, often the first to colonize the coastal alluvium of lakes, rivers, and streams, as well as abandoned farmland and rocky outcrops [5,6].

The species plays a significant role in flood control, riverbank stabilization, and maintaining river ecosystem functionality, while also contributing to water filtration and purification [7,8]. Its wood is used in energy production; as fiber for paper and chipboard; and, most profitably, in joinery, where it is employed as solid wood or veneer. However, due to land clearance activity for agriculture, the abundance of *A. glutinosa* has been declining [9].

Biodiversity loss is a major global concern, and wild plants, as sources of genetic resources, are critically important to national economies. *A. glutinosa*, which provides vital ecological functions, is a red-listed species in Kazakhstan [10]. The species does not have a wide distribution in the country, and its populations are isolated and scattered. Notably, one of the easternmost edges of *A. glutinosa* distribution is in the Bayanaul Mountains, which is in the eastern part of Kazakhstan [11]. The population is likely more isolated than others. Additionally, the species is present in the Karkarala and Ereimentau mountain forest massifs (both in the central part of the country) and along the Ilek [12] and Ural Rivers [13] (in the western region of Kazakhstan). It is also found in the northern Turgay strata, a vast steppe plain [14]. Such fragmented populations suggest potential isolation and limited gene flow, which is important to consider when examining the genetic structure of the populations, particularly in comparison with populations in the western and central parts of the range of the species.

Understanding the genetic diversity of *A. glutinosa* populations is essential for its conservation and breeding efforts [8,15,16]. Forest trees often exhibit high genetic diversity due to their large geographic ranges, outcrossing systems, and seed dispersal mechanisms, which include wind and animals [8]. In such a context, it is critical to assess the genetic diversity of natural populations of *A. glutinosa*. However, previous studies on the species in Kazakhstan have been limited to phenotypic traits [17,18,19], with no reports on the use of molecular markers to investigate population variability. Consequently, the lack of molecular data restricts efforts to protect, conserve, and exploit the species effectively.

Molecular markers have become invaluable tools for building *A. glutinosa* germplasm collections, evaluating local diversity, and studying population structure. Simple sequence repeats (SSRs) are widely distributed in genomes, often highly polymorphic, co-dominant, stable, and highly reproducible, making them ideal for genetic studies in woody plants such as poplar, Coffea, and Brazilian cherry [20,21,22].

Earlier work by Mingeot et al. [6] demonstrated that 11 out of 26 SSR primers produced amplification products suitable for studying the genetic structure of natural *A. glutinosa* populations. Subsequently, Lepais et al. [23] developed and validated 12 polymorphic loci for use in population genetics studies of *A. glutinosa*. In addition, using SSR primers designed by Mingeot [15], a panel of 14 nuclear SSR loci revealed high allelic diversity and very low differentiation among wild populations of *A. glutinosa* (Fst = 0.014). Martín et al. [24] also assessed the local genetic diversity of populations and identified potential hybrids resulting from the coexistence of *A. glutinosa* and *Alnus lusitanica* in Spain. In recent decades, numerous studies have investigated the genetic variability of various *Alnus* species, including *A. glutinosa* [8,15,16,24,25], *Alnus cremastogyne* [26], *Alnus incana* [27], and *A. lusitanica* [28].

In the present study, the genetic diversity and structure of *A. glutinosa* populations from the eastern part of its natural distribution range were analyzed using 12 SSR markers (Ag01, Ag05, Ag09, Ag10, Ag13, Ag14, Ag20, Ag23, Ag25, Ag27, Ag30, and Ag35). The research aimed to assess intra- and inter-population variability of black alder across different ecological conditions. This is the first molecular-level investigation of *A. glutinosa* in northern Kazakhstan, which could provide insights into local diversity and the genetic structure of the endangered species.

## 2. Results

### 2.1. Genetic Diversity of A. glutinosa Populations

The genetic diversity of natural Alnus glutinosa populations was assessed using 12 microsatellite loci: Ag01, Ag05, Ag09, Ag10, Ag13, Ag14, Ag20, Ag23, Ag25, Ag27, Ag30, and Ag35. The analysis revealed a high level of genetic diversity. The amplification of 78 samples from seven populations yielded a total of 76 alleles across 12 SSR loci (Table S1). Markers Ag01 and Ag05 displayed high genetic diversity, with Ag05 exhibiting the highest observed heterozygosity (0.837) and gene flow (Nm = 3.499), indicating significant population mixing. Ag09 also showed good diversity with adequate gene flow (Nm = 2.511). In contrast, Ag10 had lower diversity and gene flow (Nm = 0.850), suggesting restricted migration. Ag13 and Ag14 exhibited strong genetic diversity; however, Ag14’s low heterozygosity (0.304) could indicate selection or drift. Ag20 had very low diversity but high gene flow (Nm = 5.807), indicating population homogeneity. Other markers, such as Ag23 and Ag25, showed moderate diversity, while Ag27 and Ag30 demonstrated both good diversity and strong gene flow. Ag35 also showed strong diversity with moderate gene flow. Therefore, the average number of alleles per locus was 6.3, and the effective number of alleles per locus was 2.783. The observed heterozygosity (Ho = 0.570) was not significantly different from the expected heterozygosity (He = 0.562).

In total, 76 alleles were identified across the seven populations, 13 of which were private alleles (Table 1). The unique alleles were detected in five out of the seven populations: KS1, PVL3, PVL4, PVL5, and PVL6. In the KS1 population (northern Turgay), eight unique alleles were observed at loci Ag01, Ag05, Ag13, Ag14, Ag20, and Ag35, and they were found exclusively within this population. Similarly, in the Bayanaul mountain forest massif, unique alleles were detected in PVL3 (Ag13), PVL4 (Ag14), PVL5 (Ag20, Ag30), and PVL6 (Ag35).

Genetic diversity across the seven populations of *A. glutinosa* is summarized in Table 2. The KS1 population (northern Turgay) exhibited the highest Na value (5.000), while the PVL6 population (Bayanaul mountain forest massif) had the lowest (Na = 3.583). In terms of effective alleles, KS1 had the highest Ne (3.246), whereas PVL5 (Bayanaul mountain forest massif) had the lowest Ne (2.469). Ho across populations ranged from 0.491 to 0.681, while He varied from 0.504 to 0.634, with overall mean values of 0.570 and 0.562, respectively. Slightly higher uHe values compared to Ho were observed across all seven populations, suggesting that random mating is the dominant system, with limited inbreeding within *A. glutinosa* populations. Although uHe values were consistently higher than Ho values across all seven populations, the considerable variation in sample size (N) calls for caution when interpreting the differences. The elevated uHe values may reflect the predominance of random mating and limited inbreeding within *A. glutinosa* populations. However, the trend is likely influenced by the disparity in sample sizes across the studied populations.

Unbiased expected heterozygosity (uHe) and Ho ranged from 0.029 to 0.761 and 0.029 to 0.837, respectively, with mean values of 0.592 and 0.570 (Table 2). The Ag05 and Ag35 loci exhibited the highest uHe (0.761 and 0.759, respectively), as well as the greatest number of different alleles (Na = 9) and effective alleles (Ne = 3.675 and 3.888, respectively). Among the 12 SSR loci, Ag14 had a lower Ho than uHe, while Ag01, Ag05, Ag09, Ag10, Ag27, and Ag30 exhibited slightly higher Ho compared to uHe. Overall, the mean Ho was nearly equal to the mean uHe, indicating a predominantly random mating system within *A. glutinosa* populations. Shannon’s Information Index across all loci was 1.053, indicating moderate genetic diversity in the populations studied. Loci Ag05, Ag13, and Ag35 exhibited the highest diversity, whereas Ag10 and Ag20 had the lowest diversity, reflecting differences in allele distribution and heterozygosity among the loci.

Shannon’s Information Index (I) across populations indicated varying levels of genetic diversity, with KS1 showing the highest diversity and PVL5 the lowest. The average I across all populations was 1.053, suggesting moderate overall genetic diversity in the studied *A. glutinosa* populations, which is consistent with the range of effective allele counts and observed heterozygosity values across the populations.

In conclusion, the overall genetic diversity within *A. glutinosa* populations was relatively high, with the KS1 (northern Turgay) and PVL3 (Bayanaul mountain forest massif) populations exhibiting the greatest diversity. In contrast, the PVL5 population (Bayanaul mountain forest massif) showed slightly lower levels of genetic diversity.

### 2.2. Genetic Differentiation of A. glutinosa Populations

Genetic differentiation among the seven *A. glutinosa* populations, measured based on *G*’st (Nei), ranged from 0.217 (Ag10) to −0.003 (Ag20) across the 12 loci, with a mean of 0.078 (Table S2). This indicates that 7.8% of the genetic variation existed among the populations, while the remaining 92.2% was found within populations, highlighting that genetic variation within populations was the primary source of overall diversity.

Nm across the 12 SSR loci averaged 2.565, which is slightly higher than 1, aligning with the moderate level of genetic differentiation observed among populations (Table S2). This suggests a moderate degree of gene flow among the seven *A. glutinosa* populations. Additionally, Ho was generally similar to He or uHe in all populations, further supporting moderate heterozygosity levels.

Positive values for Wright’s F-statistics confirm sufficient heterozygosity at the population level (Table S2). The FST values ranged from 0.041 at the Ag20 locus to 0.227 at the Ag10 locus, with an average FST of 0.110, indicating significant genetic differentiation between populations.

Pairwise comparisons of populations (Table S3) revealed that the greatest contribution to this high interpopulation differentiation stemmed from differences between population KS2 (northern Turgay) and populations PVL3, PVL4, PVL5, and PVL7 (Bayanaul mountain forest massif). Population KS2, located in the Kostanay region, is approximately 1020 km away from the Pavlodar region populations (PVL3, PVL4, PVL5, and PVL7), highlighting the influence of geographic distance on genetic differentiation.

The pairwise F_ST_ values for these populations ranged from 0.041 to 0.106, with an average F_ST_ of 0.068. Among the pairwise comparisons, the highest F_ST_ (0.106) was found between populations KS2 (northern Turgay) and PVL3 (Bayanaul mountain forest massif), while the lowest F_ST_ (0.041) was observed between PVL4 and PVL7 (both in the Bayanaul mountain forest massif). Correspondingly, gene flow (Nm) ranged from 0.850 to 5.807, with most values exceeding 1, indicating a relatively high level of gene flow between paired populations.

**Figure 1:**
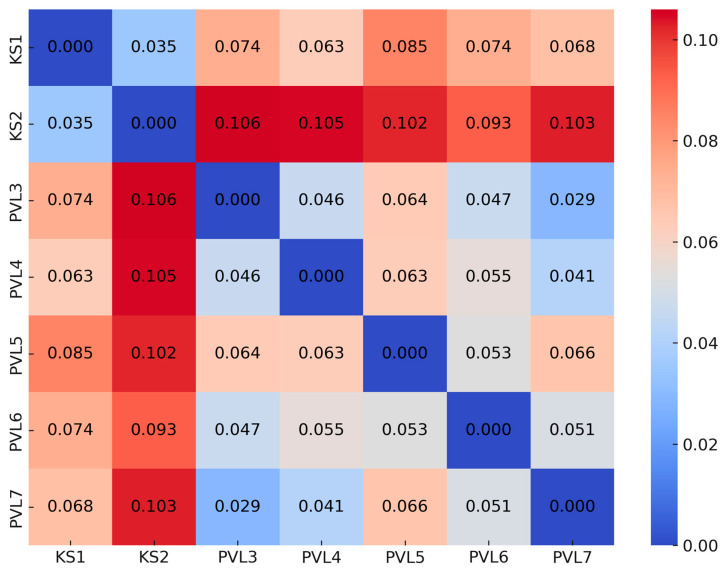
Heatmap of paired Fst values among *A. glutinosa* populations.

The heatmap (Figure 1) reveals moderate genetic differentiation among *A. glutinosa* populations. The relatively low Fst values between PVL populations suggested greater genetic cohesion, whereas the higher values between KS populations and PVL populations highlighted greater genetic differentiation. Understanding the trends is critical for formulating effective conservation and management strategies to ensure the preservation of genetic diversity in the species.

**Figure 2:**
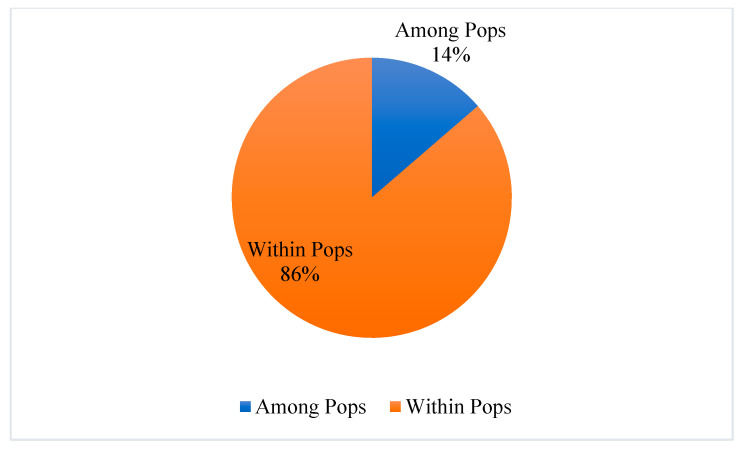
Results of analysis of molecular variance (AMOVA) of seven populations of *A. glutinosa*.

An analysis of molecular variance (AMOVA) conducted using 12 SSR loci (Figure 2) revealed that 86% of the genetic variation was within populations, while 14% was between populations (summarizing table available in Supplementary Table S3). This result aligns with the *G*’st (Nei) value of 0.078 calculated from the F-statistic (Table S1), reinforcing the conclusion that the majority of genetic variability in *A. glutinosa* exists within populations.

### 2.3. Genetic Structure of the A. glutinosa Population

**Figure 3:**
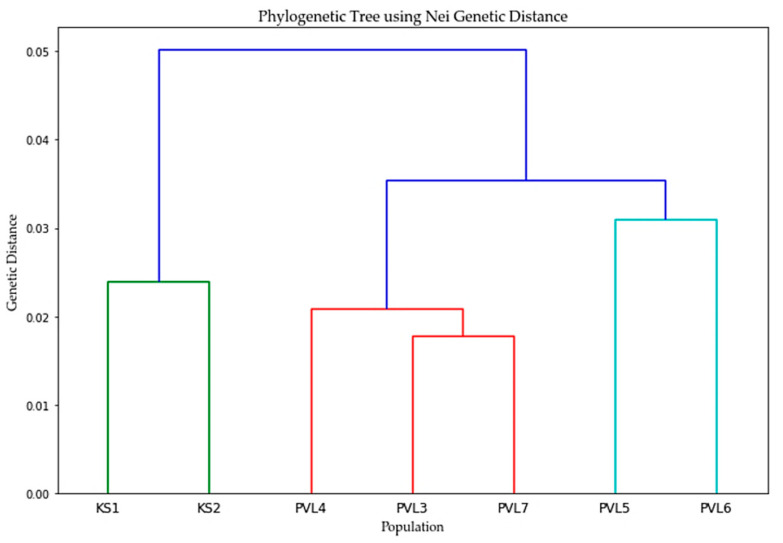
Population genetic structure: UPGMA cluster analysis of seven *A. glutinosa* populations based on Nei’s genetic distance.

The UPGMA tree constructed based on Nei’s genetic distance values revealed that the seven populations of *A. glutinosa* could be divided into three distinct clusters: (KS1, KS2); (PVL4, PVL3, and PVL7); and (PVL5, PVL6). The KS1 and KS2 populations were located in northern Turgay (Kostanay region), while the PVL3, PVL4, PVL5, PVL6, and PVL7 populations were from the Bayanaul mountain forest massif (Pavlodar region). This clustering pattern suggests that the genetic variation in *A. glutinosa* populations is geographically structured. The second cluster can be divided further into two subgroups: one consisting solely of PVL4 and the other comprising PVL3 and PVL7. However, the subgroups did not exhibit clear geographical differentiation (Figure 3).

**Figure 4:**
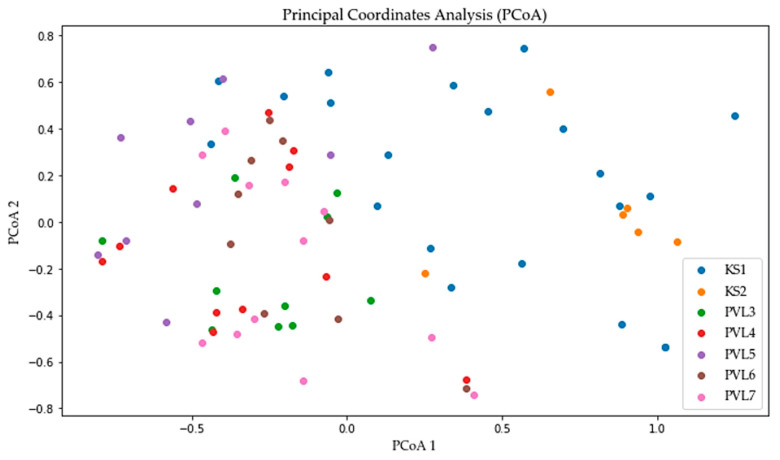
Principal coordinate analysis of seven *A. glutinosa* populations.

Principal coordinate analysis (PCoA) was conducted to visualize the genetic relationships among seven *A. glutinosa* populations, labeled as KS1, KS2, PVL3, PVL4, PVL5, PVL6, and PVL7 (Figure 4). Each point on the plot represents an individual, and the clustering of points indicates genetic similarity. Populations KS1 (blue) and KS2 (orange) showed distinct separation from the other populations along the first principal coordinate, indicating substantial genetic differentiation. In contrast, populations PVL3, PVL4, PVL5, PVL6, and PVL7 exhibited greater overlap, suggesting that the populations had more genetic similarities or had experienced gene flow. The second principal coordinate (PCoA 2) accounted for additional variation, but the most prominent genetic separation was along the first coordinate.

Overall, the results highlighted significant genetic diversity among some populations, particularly KS1 and KS2, while the others were more genetically interconnected. The information could facilitate the formulation of conservation strategies and understanding of population structure within the species.

## 3. Discussion

### 3.1. Population Genetic Structure and Geographical Variation in A. glutinosa

This study investigated the genetic diversity of seven *A. glutinosa* populations growing in two regions of northern Kazakhstan: the Kostanay and Pavlodar regions. In the northern Turgay (Kostanay region), 27 trees were sampled from two populations (KS1 and KS2), while 51 trees were sampled from five populations in the Bayanaul mountain forest massif (Pavlodar region; PVL3, PVL4, PVL5, PVL6, and PVL7).

To assess genetic diversity, we used 12 nuclear microsatellite markers. The results are consistent with the findings of previous studies that have employed microsatellites or other DNA markers to evaluate genetic diversity and hybridization within *A. glutinosa* populations [8,15,16,24,25].

Our findings reveal significant genetic differentiation and structure among the two regional populations, as well as within the populations in each region. Both regions exhibited comparable levels of genetic variation, with a large number of common alleles and several unique alleles.

All seven *A. glutinosa* populations demonstrated relatively high genetic diversity, with Na = 6.3 and uHe = 0.592 across the 12 SSR loci. Populations KS1 (northern Turgay) and PVL3 (Bayanaul mountain forest massif) showed the highest levels of genetic diversity.

The genetic diversity observed in Kazakhstan’s *A. glutinosa* populations was comparable to that in populations from Latvia (Na = 10.07, uHe = 0.636) [16], as well as the genetic diversity in Irish, Scottish, and French populations (Na = 6.61, He = 0.64) [8]. Additionally, the genetic diversity of populations along the borders of Belgium, Luxembourg, and France (Na = 7.34, He = 0.64) [15], as well as that of *A. cremastogyne* (Na = 5.83, He = 0.630) [26], as determined by SSR markers, was similar to that of *A. glutinosa*.

The FST value for *A. glutinosa* (Fst = 0.110) indicates relatively high genetic differentiation, which is higher than that observed in *A. glutinosa* populations on the Belgium–Luxembourg–France border (Fst = 0.014) [28] and in *A. cremastogyne* populations (Fst = 0.021) [25] but similar to *A. maritima* populations (Fst = 0.107) [29]. This elevated differentiation could be attributed to the fragmented and isolated nature of the eastern populations, which likely restricts gene flow. Nonetheless, the gene flow estimate (Nm = 2.565) suggests moderate levels of gene flow among populations, though this may vary depending on the degree of population isolation across the species’ range.

The classification of *A. glutinosa* populations into three clusters supports the presence of geographically structured genetic variability. Cluster 1 includes the populations from northern Turgay (KS1, KS2), which also show distinct separation from other populations in the PCoA, indicating substantial genetic differentiation. Cluster 2 comprises populations PVL3, PVL4, and PVL7 from the Bayanaul mountain forest massif, and Cluster 3 includes populations PVL5 and PVL6 from the same region. Interestingly, while the PCoA reveals genetic overlap among the Bayanaul populations, the cluster analysis separates them into two groups. This genetic overlap may be due to the species’ preference for riparian habitats and the lightweight nature of its seeds, which facilitate dispersal via regional water systems, such as the Mayozek River, Lake Toraigyr, and various springs. The PCoA results support the view that gene flow has occurred among these, despite geographic distances (Figure 1). *A. glutinosa* seeds are dispersed primarily by running water rather than wind, with dispersal typically limited to around 30 m from the parent trees [30]. Consequently, the species tends to form linear stands along streams and rivers.

Additionally, significant isolation by altitude was observed in the genetic structure of the *A. glutinosa* populations. As the altitudinal differences between populations increased, genetic distance also increased. Populations in Cluster 2 (PVL3, PVL4, and PVL7) were situated along a similar altitudinal gradient, while those in Cluster 3 (PVL5 and PVL6) were located at lower elevations (Table S1). The heatmap (Figure 1) shows moderate genetic differentiation between populations, with lower Fst values among PVL populations, indicating higher genetic cohesion. In contrast, higher Fst values between KS and PVL populations highlight greater genetic differentiation, likely due to geographic isolation. The patterns emphasize the need to account for geographic barriers in conservation strategies.

In conclusion, positive correlations between genetic and geographic distances, as evidenced by the statistically significant Mantel test result (Mantel statistic = 1.0000, *p*-value = 0.0010), suggest that the observed genetic differentiation could be attributed to isolation by both distance and altitude. This implies that geographical separation, possibly amplified by altitudinal barriers, has likely limited gene flow between populations, leading to the patterns of genetic diversity observed in the present study.

### 3.2. Genetic Improvement of A. glutinosa

*A. glutinosa* is one of the most important forest tree species globally, thriving in damp habitats and exhibiting significant sensitivity to fluctuations in groundwater levels. Its seeds are dispersed primarily by water, leading to the species’ characteristic growth in linear stands along streams and rivers. However, the distribution exhibits no specific correlation with river catchments.

Human activity is increasingly disrupting the natural habitats of *A. glutinosa*, posing a significant threat to its survival. Despite its high potential for long-distance seed dispersal via water, the species’ expansion is constrained by its intolerance to shade, which limits seed germination and seedling survival in areas already populated by other trees [9,15].

Additionally, *A. glutinosa* faces a severe threat from the fungal disease *Phytophthora alni*, which has led to significant die-offs and population declines across Europe [6,31].

Considering the advanced stage of the disease, in situ conservation has become increasingly challenging, making the conservation of the remaining populations imperative. To protect *A. glutinosa*, priority should be given to ex situ conservation efforts, including the establishment of germplasm collections from the most genetically significant populations across different ecological zones. This can be achieved through vegetative propagation, micropropagation, and cryopreservation of seeds [32,33,34]. Populations such as KS1, PVL3, PVL4, PVL5, and PVL6, which exhibit the highest genetic diversity, should be prioritized for ex situ conservation efforts.

## 4. Materials and Methods

### 4.1. Plant Material

**Figure 5:**
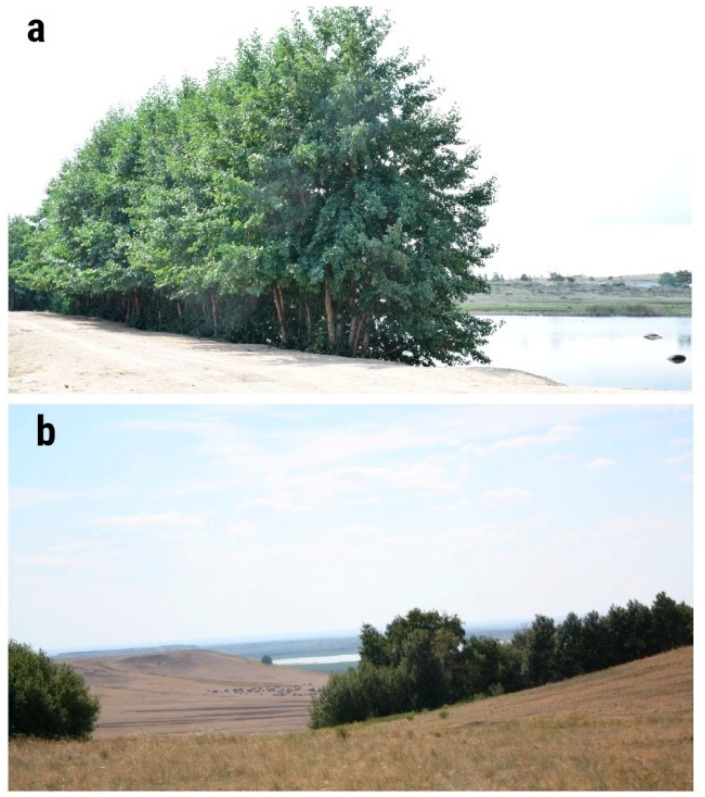
*A. glutinosa* growing in the Bayanaul mountain forest massif and northern Turgay of northern Kazakhstan: (**a**) *A. glutinosa* growing in the Bayanaul mountain forest massif; (**b**) *A. glutinosa* growing in northern Turgay.

The study area encompassed the northern Kazakhstan Bayanaul mountain forest massif (Pavlodar region) and northern Turgay (Kostanay region). A total of seven natural populations of alder forests were sampled, consisting of five populations from the Bayanaul mountain forest massif and two populations from northern Turgay, which were located along riverbanks (Table 3, Figure 5). Plant material was collected from 78 trees across the regions. Within each population, samples were collected with a minimum spacing of 15 m between trees.

**Figure 6:**
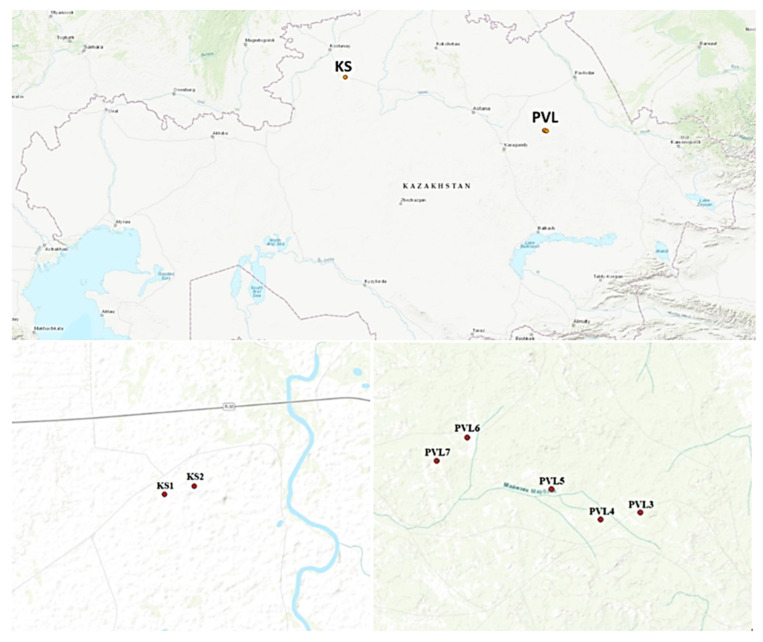
Collection site of seven populations of *A. glutinosa* in the Bayanaul mountain forest massif and northern Turgay in northern Kazakhstan.

The geographic coordinates and elevation of each specimen were recorded using a Garmin GPS MAP 60 CX Navigator (Olathe, KS, USA). The locations of the populations are displayed in Figure 6. Trees within each population were sequentially numbered. Leaf samples were gathered, transported to the laboratory, and stored at −30 °C until further analysis.

### 4.2. DNA Extraction and Amplification

Genomic DNA was extracted using a modified CTAB method [35]. The concentration and purity of the extracted DNA were evaluated using a NanoDrop 1000 ultraviolet spectrophotometer (Thermo Scientific, Wilmington, NC, USA). The study employed SSR primers developed by Lepais et al. [24], as detailed in Table 2. PCR reactions were conducted in a total volume of 25 µL, which included 60 ng of template DNA, 12.5 µL of BioMaster HS-qPCR (2×) (Biolabmix, Novosibirsk, Russia), 1 µL of each primer (10 pmol), and 7.5 µL of ddH_2_O. PCR amplification was carried out in a SimpliAmp™ Thermal Cycler (Thermo Fisher Scientific Inc., Waltham, MA, USA) with the following cycling parameters: initial denaturation at 95 °C for 5 min; followed by 30 cycles of 95 °C for 20 s, 58 °C for 3 min, and 72 °C for 30 s; with a final extension at 60 °C for 30 min.

The amplified fragments were separated on an ABI3730xl automated genetic analyzer (Applied Biosystems, Tokyo, Japan) using POP7 polymer and GeneScan™ 500 LIZ^®^ size standard. Fragment sizes were analyzed using GeneMapper 6.5 software (Applied Biosystems). The sizing of the amplified fragments was performed with GeneMarker^®^ v2.2.0 software (SoftGenetics, State College, PA, USA). The forward primers used in the study were labeled fluorescently, as listed in Table 4. We used two multiplex mixes for PCR amplification. Mix 1 included the following loci: Ag05, Ag09, Ag10, Ag13, Ag14, Ag20, Ag25, and Ag30. Mix 2 comprised the following loci: Ag01, Ag23, Ag27, and Ag35. The device was calibrated for the corresponding dyes using the CS5 matrix standard (COrDIS, Russia).

### 4.3. Data Analysis

To characterize the 12 SSRs and assess the genetic diversity, the GenAlex 6.5 software [36], operating within MS Excel (Microsoft Corp., Redmond, WA, USA), was employed. The genetic diversity and structure of *A. glutinosa* across the seven populations were assessed using the following parameters: total number of alleles (Na) per locus, effective number of alleles (Ne), observed heterozygosity (Ho), expected heterozygosity (He), unbiased expected heterozygosity (uHe), gene flow (Nm), Nei’s genetic differentiation (G’st), and unique alleles across the populations. The genetic diversity of the 78 sampled trees was further analyzed by calculating additional metrics across the seven populations, including sample size (N), allelic diversity (Na), the effective number of alleles (Ne), heterozygosity measures (Ho, He, and uHe), and Shannon’s Information Index (I).

Population differentiation was evaluated using F-statistics across the 12 SSR loci to quantify the proportion of genetic variance among populations relative to the total variance. An AMOVA was conducted to partition the genetic variance within and among the seven populations, providing insight into the distribution of genetic diversity. To further examine the genetic structure and relationships among the populations, a UPGMA phylogenetic tree was constructed based on Nei’s genetic distance. The tree was generated using the matplotlib.pyplot and scipy.cluster.hierarchy libraries in Python. Genetic analyses were performed using Python libraries. A heatmap of pairwise Fst values between populations was generated using seaborn and customized with matplotlib. Principal Coordinate analysis (PCoA) was conducted using scikit-learn for dimensionality reduction, and plots were created with matplotlib. The Mantel test, assessing the correlation between genetic and geographic distances, was performed with the pingouin library using 999 permutations.

## 5. Conclusions

This study represents the first investigation of *A. glutinosa* populations in Kazakhstan using SSR markers to assess genetic diversity and structure. The findings reveal that the seven *A. glutinosa* populations exhibit a high level of genetic diversity and relatively strong gene flow among them. The populations are grouped into three clusters, each reflecting distinct geographic distribution patterns. The insights gained from this study could contribute to the conservation efforts and formulation of genetic enhancement strategies for *A. glutinosa*, a species of significant ecological importance listed in the Red Book of Kazakhstan.

## Figures and Tables

**Table 1:** Number of individuals carrying unique alleles by population.

Locus	Allele Size	Population				
		KS1	PVL3	PVL4	PVL5	PVL6
Ag01	135	2	-	-	-	-
Ag05	159	1	-	-	-	-
Ag13	259	5	-	-	-	-
	283	-	1	-	-	-
Ag14	293	10	-	-	-	-
	295	1	-	-	-	-
	315	2	-	-	-	-
	325	-	-	2	-	-
Ag20	311	-	-	-	1	-
	313	2	-	-	-	-
Ag30	102	-	-	-	1	-
Ag35	183	-	-	-	-	1
	209	3	-	-	-	-

**Table 2:** Genetic diversity of 78 *A. glutinosa* trees representing seven populations in northern Turgay and Bayanaul mountain forest massif based on 12 microsatellite loci.

Population	N	Na	Ne	Ho	He	uHe	I
KS1	21	5.000	3.246	0.583	0.634	0.649	1.248
KS2	6	3.667	2.629	0.681	0.542	0.591	0.998
PVL3	10	3.667	2.834	0.533	0.586	0.617	1.068
PVL4	11	3.750	2.823	0.561	0.554	0.580	1.038
PVL5	9	3.667	2.469	0.491	0.504	0.534	0.948
PVL6	9	3.583	2.708	0.583	0.557	0.589	1.019
PVL7	12	3.917	2.775	0.556	0.558	0.583	1.051
Mean	11.143	3.893	2.783	0.570	0.562	0.592	1.053

N: number of alleles; Na: number of different alleles; Ne: number of effective alleles; Ho: observed heterozygosity; He: expected heterozygosity; uHe: unbiased expected heterozygosity; I: Shannon’s Information Index.

**Table 3:** Locations and numbers of trees sampled in seven *A. glutinosa* populations.

Population	Location	Latitude (N)	Longitude (E)	Elevation (m)	Sample Size
KS1	Northern Turgay	52°32′39″	64°46′46″	130–160	14
KS2		52°32′22″	64°45′44″	120–160	13
PVL3	Bayanaul mountain forest massif	50°48′354″	75°42′893″	483–498	10
PVL4		50°49′595″	75°39′729″	477–504	11
PVL5		50°50′745″	75°32′345″	381–388	9
PVL6		50°51′698″	75°34′315″	401–406	9
PVL7		50°48′640″	75°45′450″	459–467	12

**Table 4:** Characteristics of 12 microsatellite primers (SSRs) used in this study.

Loci	Primer Sequences	Dye
Ag01	F: CAGTCTATCTGCTACAAGCGTGGT	FAM
	R: GACGTTTTCAACGACCAAAAACAC	
Ag05	F: AAGCAAAATCCCAAGGTATCCAGT	FAM
	R: GGGGTTCCAACCAATTTATTCTTC	
Ag09	F: GATGGTAATGTGACGTGAGCAAAA	ROX
	R: CCTATTCTCATCGTTTAAAGCCCC	
Ag10	F: AACTTGTCTTATTGTGCACTTGCG	FAM
	R: ACATTTACGGCTAAACAGCATTCC	
Ag13	F: CAAGCGAAATAGATTCGTGGTCTT	R6S
	R: CTTCCATTTGGAGCCTTAAAACAC	
Ag14	F: CAACCAACAAGGAGACAGAAACAA	FAM
	R: TAAAATCTAACACCCCAAACGAGG	
Ag20	F: GGTTCCAAGTGGTAAGGGGAGTTA	TAMRA
	R: GAGTGTGAGAATGTGGTTCACGAG	
Ag23	F: GGTTGGGCGAAAGTTTTATTTACAC	R6G
	R:CCAGAAACGAACTAAGGCTAAGAAGA	
Ag25	F: GGATAAGAAGATAAAGGTGCATGGC	ROX
	R: CTGTATATCCCCACCACACCTGA	
Ag27	F: CATTTGGTGTATGTGTTGCCAGTT	ROX
	R: AATCAACAACTGCCAGGTAGAGGA	
Ag30	F: GGAACTCTGGAAACAGAAACAACG	FAM
	R: AGCAAGGTAAAACTTCAGTAGCCG	
Ag35	F: CACGTTCAGCTTCATTGTGACTTC	ROX
	R: TAATAGGGTTTGGGCCAACTTACC	

## Data Availability

The data presented in this study are available on request from the corresponding author.

## Supplementary Materials

The following supporting information can be downloaded at: https://www.mdpi.com/article/10.3390/plants13213032/s1, Table S1: Characterization of 12 simple sequence repeat loci in Alnus glutinosa based on 78 trees representing 7 populations, Table S2: F-statistics for all populations for each locus, Table S3: Summary AMOVA (Analysis of Molecular Variance) table for the populations studied.
