# Supplementary material for: Assessing Genetic Variability and Population Structure of Alnus glutinosa (Black Alder) in Kazakhstan Using SSR Markers
Source: Plants (Basel). 2024 Oct 30;13(21):3032. doi: 10.3390/plants13213032 (PMC11548218; doi:10.3390/plants13213032)
Supplement: Supplementary file 1 [file plants-13-03032-s001.zip › plants-3201369-supplementary.pdf]

Table S1. Characterization of 12 simple sequence repeat loci in *A. glutinosa* based on 78 trees representing 7 populations.

Locus	Size Range	Na	Ne	Ho	He	uHe	I	G'st(Nei)	Nm	*
Ag01	129-145	7	3.608	0.826	0.711	0.748	1.404	0.076	2.187	135
Ag05	145-163	9	3.675	0.837	0.722	0.761	1.433	0.033	3.499	159
Ag09	244-256	7	3.098	0.733	0.663	0.699	1.243	0.059	2.511	-
Ag10	217-233	4	1.599	0.327	0.304	0.321	0.515	0.217	0.850	-
Ag13	255-283	10	3.471	0.724	0.696	0.734	1.358	0.139	1.301	259, 283
Ag14	291-325	10	3.473	0.304	0.699	0.736	1.343	0.034	2.301	293, 295, 315, 325
Ag20	309-313	3	1.031	0.029	0.028	0.029	0.058	-0.003	5.807	311, 313
Ag23	354-360	4	2.344	0.561	0.563	0.593	0.958	0.072	2.107	-
Ag25	101-110	3	1.821	0.454	0.431	0.453	0.664	0.160	1.150	-
Ag27	107-115	4	2.324	0.619	0.545	0.574	0.982	0.015	4.471	-
Ag30	102-112	6	3.069	0.790	0.660	0.695	1.203	0.059	2.625	102
Ag35	179-209	9	3.888	0.632	0.720	0.759	1.474	0.074	1.966	183, 209
Mean		6.3	2.783	0.570	0.562	0.592	1.053	0.078	2.565	
Na: Number of different alleles; Ne: Number of effective alleles; Ho: Observed heterozygosity; He: Expected Heterozygosity; uHe: = Unbiased Expected Heterozygosity; F = Fixation Index; I = Shannon's Information Index; G'st(Nei) = Nei's standardized Gst; Nm: Gene flow; *: Unique alleles characteristic of only one population										

Table S2. F-statistics for all populations for each locus.

Locus	F-statistics		
	Fis	Fit	Fst
Ag01	-0.162	-0.043	0.103
Ag05	-0.158	-0.081	0.067
Ag09	-0.105	-0.005	0.091
Ag10	-0.076	0.168	0.227
Ag13	-0.039	0.128	0.161
Ag14	0.565	0.608	0.098
Ag20	-0.055	-0.011	0.041
Ag23	0.002	0.108	0.106
Ag25	-0.053	0.135	0.179
Ag27	-0.135	-0.075	0.053
Ag30	-0.196	-0.092	0.087
Ag35	0.123	0.222	0.113
Mean	-0.024	0.089	0.110

Table S3. Summary AMOVA Table

**Source**	**df**	**SS**	**MS**	**Est. Var.**	**%**
**Among Pops**	6	124,961	20,827	1,213	14%
**Within Pops**	71	544,103	7,663	7,663	86%
**Total**	77	669,064		8,876	100%
